# Benign lesions that mimic cancer: Ovarian

**DOI:** 10.1186/1470-7330-15-S1-O22

**Published:** 2015-10-02

**Authors:** Rosemarie Forstner, Thomas Meissnitzer, Matthias Meissnitzer

**Affiliations:** 1 Department of Radiology, Landeskliniken Salzburg, PMU, Salzburg, 5020 Austria

## 

As ovarian masses are common in both pre- and in postmenopausal age, a life time risk of up to 5-10% to undergo pelvic surgery has been reported [1]. However, the likelihood of malignancy in these lesions is extremely low. Imaging, particularly, US and MRI, have been integrated as diagnostic tools to better define indications for adequate surgery and to guide referral to specialized centers. Imaging findings are assessed in the context with clinical data, patient history, age, and tumor markers. Furthermore, various risk assessment indices to predict malignancy have been established using clinical and/or imaging parameters.

Advances in imaging including the combination of morphologic and functional parameters have further improved the diagnostic performance of MRI. Thus the majority of indeterminate masses on US and CT can be correctly diagnosed with MRI [2,3]. MRI is most beneficial in women with a low likelihood of cancer. Endometriomas, common mimicks of ovarian cancer in CT and US, display specific imaging findings in MRI. This is also true for subserous leiomyomas, which are often difficult to differentiate from solid ovarian masses in US. Meticulous analysis of anatomical landmarks and displacement patterns aid in differentiation of benign extraovarian tumors, particulary of extraperitoneal origin, e.g of neurinomas, from ovarian cancer. Some complex cystic and solid adnexal masses may be challenging to differentiate from ovarian cancer. In these lesions integration of clinical findings will allow in most cases differentiation of benign lesions from cancer. Ovarian torsion, pelvic hematoma, and extrauterine pregnancy are typically associated with pelvic pain. Pain and inflammatory laboratory findings suggest tubo ovarian abscess (TOA). TOA may mimick ovarian cancer even in advanced imaging, including MRI and PET/CT. Aspergillosis, a subtype of TOA mimicks ovarian cancer due to its complex morphology and invasive growth pattern. Differentiation of peritoneal tuberculosis from advanced ovarian cancer is difficult and requires biopsy [4].

Ovarian pseudocysts and cystadenofibroma display typical findings in most cases. However, some of the benign pelvic lesions still present a diagnostic dilemma [5]. These include rare entities, e.g. atypical dermoids, monodermal dermoids, collision tumors, and sometimes also hemorrhage of functional cysts.
